# Engineered Microstructure Derived Hierarchical Deformation of Flexible Pressure Sensor Induces a Supersensitive Piezoresistive Property in Broad Pressure Range

**DOI:** 10.1002/advs.202000154

**Published:** 2020-08-19

**Authors:** Gang Li, Duo Chen, Chenglong Li, Wenxia Liu, Hong Liu

**Affiliations:** ^1^ State Key Laboratory of Biobased Materials and Green Papermaking Qilu University of Technology Shandong Academy of Science Jinan Shandong 250353 China; ^2^ Institute for Advanced Interdisciplinary Research University of Jinan (iAIR) Jinan 250022 China; ^3^ State Key Laboratory of Crystal Materials Shandong University Jinan 250100 P. R. China

**Keywords:** engineered microstructures, flexible tactile sensors, hierarchical deformation modes, highest sensitivity, piezoresistive pressure sensors

## Abstract

Fabricating flexible pressure sensors with high sensitivity in a broad pressure range is still a challenge. Herein, a flexible pressure sensor with engineered microstructures on polydimethylsiloxane (PDMS) film is designed. The high performance of the sensor derives from its unique pyramid‐wall‐grid microstructure (PWGM). A square array of dome‐topped pyramids and crossed strengthening walls on the film forms a multiheight hierarchical microstructure. Two pieces of PWGM flexible PDMS film, stacked face‐to‐face, form a piezoresistive sensor endowed with ultrahigh sensitivity across a very broad pressure range. The sensitivity of the device is as high as 383 665.9 and 269 662.9 kPa^−1^ in the pressure ranges 0–1.6 and 1.6–6 kPa, respectively. In the higher pressure range of 6.1–11 kPa, the sensitivity is 48 689.1 kPa^−1^, and even in the very high pressure range of 11–56 kPa, it stays at 1266.8 kPa^−1^. The pressure sensor possesses excellent bending and torsional strain detection properties, is mechanically durable, and has potential applications in wearable biosensing for healthcare. In addition, 2 × 2 and 4 × 4 sensor arrays are prepared and characterized, suggesting the possibility of manufacturing a flexible tactile sensor.

## Graphical abstract

A flexible piezoresistive pressure sensor with hierarchical microstructures on its interlocked polydimethylsiloxane sensing films is fabricated by using a specially designed silicon template with the unique pyramid‐wall grid microstructures of different height and a four‐step mold‐casting process. The sensor has ultrahigh sensitivity in a very wide pressure range, yielding better performance than most flexible pressure sensor in literature.

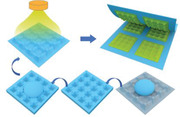

## 1 Introduction

Flexible pressure sensors are among the most important flexible electronic devices. They can conform to the shapes of arbitrary curvilinear surfaces and produce electrical signals under the pressures generated by typical human activities, such as physical contact and the regular physiological processes of the human body.^[^ ^1^, ^2^ ^]^ The concept of an electronic skin (e‐skin) based on flexible pressure sensors with high sensitivity and a wide enough sensing range to detect the pressures produced by normal touch and object manipulation has therefore attracted a great deal of attention in such fields as artificial intelligence, human–machine interaction, health monitoring, and soft robotics.^[^ ^3^, ^4^, ^5^ ^]^ For example, it is hoped that tactile organs could give a robot the ability to feel the world by touching, to pick up and hold objects skillfully, and even to communicate or interact in a friendly way with human beings and other robots. Such intelligent interactions require high‐performance flexible e‐skins with high sensitivity and good stability.^[^ ^6^, ^7^, ^8^, ^9^, ^10^, ^11^, ^12^, ^13^ ^]^

Flexible pressure sensors have been fabricated on the basis of a variety of sensing mechanisms: piezoresistivity, piezocapacitance, piezoelectricity, and triboelectricity.^[^ ^6^, ^14^, ^15^, ^16^, ^17^, ^18^, ^19^, ^20^ ^]^ In particular, piezoresistive pressure sensors are being intensively investigated; they show great potential for practical applications owing to their ease of fabrication, simple structure, excellent sensitivity, low cost, and metal coating/nanoparticles/nanowires.^[^ ^21^, ^22^ ^]^ Among the elastomers used in such sensors, polydimethylsiloxane (PDMS) is the favorite choice because of its commercial availability and unique mechanical, chemical, and optical properties.^[^ ^23^ ^]^

PDMS films with various surface and hierarchical microstructures, both regular and irregular, have been developed. These are typically made by casting or coating onto templates, such as well‐designed silicon molds,^[^ ^24^, ^25^, ^26^, ^27^ ^]^ laser engraved molds,^[^ ^28^ ^]^ natural leaves/petals,^[^ ^29^, ^30^ ^]^ silk,^[^ ^31^ ^]^ sandpaper/abrasive paper,^[^ ^32^ ^]^ colloid self‐assembly particle film,^[^ ^33^ ^]^ and porous polystyrene template.^[^ ^34^ ^]^ The geometrical morphology and parameters of the microstructures significantly impact the contact area and localized stress of microstructure under pressure;^[^ ^36^ ^]^ using silicon and laser‐engraved molds to fabricate microstructured PDMS substrates is advantageous because it permits tailoring the geometrical shape, size, and spatial distance of the microstructures according to the customer's specifications. PDMS‐based substrates with micropillar arrays,^[^ ^26^, ^35^, ^36^ ^]^ micropyramid arrays,^[^ ^22^, ^24^, ^25^, ^27^, ^28^, ^36^ ^]^ microdome arrays,^[^ ^20^, ^36^ ^]^ microcones,^[^ ^28^ ^]^ and conical frustum‐like microstructures^[^ ^37^ ^]^ have been used in the fabrication of highly sensitive pressure sensors. It has been found that all microstructured PDMS‐based piezoresistive sensors show much higher pressure sensitivity than those based on planar substrates, due to the larger deformation of microstructured PDMS.^[^ ^20^, ^21^, ^22^, ^23^, ^24^, ^25^, ^26^, ^27^, ^28^, ^35^ ^]^

Micropillar, micropyramid, and microdome arrays are the three microstructures that have been investigated most extensively.^[^ ^36^ ^]^ Piezoresistive sensors with conical frustum‐like microstructures show higher sensitivity and a broader working range when the larger area is on top.^[^ ^37^ ^]^ Pressure sensors based on irregular microhump structures or spinosum microstructures of random distribution, i.e., micropatterns with different heights and diameters modulated from sandpaper or other abrasive paper, show higher sensitivity and a broader loading pressure range than do those based on regular micropatterns; this is due to the extra conducting paths formed by unsimultaneous contact of irregular micropatterns.^[^ ^32^, ^38^ ^]^ In addition, piezoresistive sensors with substrates of interlocked microstructures, i.e., face‐to‐face assemblies of microstructured substrates, have been found to be capable of inducing a much larger change in contact area and localized stress concentration than those with substrates of single microstructures.^[^ ^20^, ^36^ ^]^ Increasing the number of touchpoints by assembling one micropatterned substrate with another rough one, i.e., forming rough‐rough microstructures, can also significantly improve the sensitivity of pressure sensors.^[^ ^39^, ^40^ ^]^ These discoveries reveal the advantages of using tailored, interlocked/rough–rough irregular microstructures with large top areas and different heights for fabricating highly sensitive piezoresistive sensors.

However, it is difficult to tailor microstructures accurately using irregular sandpaper templates. At the same time, most of the reported pressure sensors with nano/microstructured patterns only show high sensitivity when the pressure is very low.^[^ ^38^, ^39^, ^40^ ^]^ Pressure sensors with smaller size nano/microstructures possess high sensitivity in the low pressure range but also have less stability, while those with larger‐size microstructures are mainly suitable for high pressure measurements only, but keep good stability. How to design and inexpensively manufacture flexible pressure sensors with high sensitivity in both the low and high pressure ranges is still a great challenge which must be overcome if the widespread use and mass production of pressure sensors and electronic skins is to become practicable.^[^ ^12^ ^]^

To fabricate a flexible piezoresistive pressure sensor with high sensitivity across a broad pressure range and to overcome the difficulties for tailoring irregular microstructures, we engineered a pyramid‐wall grid microstructure (PWGM) based on a hierarchical deformation mechanism by using PDMS as elastomer. The PWGM flexible PDMS film for use in a flexible piezoresistive pressure sensor is prepared by a 4‐step molding method, using a specially designed silicon microarray as a master mold. The flexible PDMS film is constituted of square pyramids possessing dome‐like heads with large top areas, together with strengthening walls connecting all the pyramids. These walls are not as high as the pyramids, and those parallel to the *y*‐axis are not as high as those parallel to the *x*‐axis. This special microstructure endows the sensor with a hierarchical deformation mechanism: when two films are interlocked, a vertical pressure will cause a series of changes in electrical resistance as first the pyramids and then the various walls come into contact and are deformed. Meanwhile, the interconnections between pyramids and walls help to increase the crushing pressure of the PWGM PDMS film. The PWGM PDMS film‐based pressure sensor based on this novel hierarchical deformation mechanism has thus been found to possess extremely high sensitivity and a fast response speed, as well as long‐term stability across a very broad pressure range. This work provides a new facile strategy for engineering pressure sensor with high sensitivity and good stability across a broad pressure range. In addition, 2 × 2, and 4 × 4 PWGM tactile sensors with micrometer resolution have been built using PWGM flexible PDMS film building blocks. Possible applications of this supersensitive pressure sensor have been explored by monitoring the author's own pulse and vocal cord vibrations, demonstrating a potential use as a multifunctional e‐skin.

## 2 Results and Discussion

### 2.1 Preparation and Characterization

**Figure 1:**
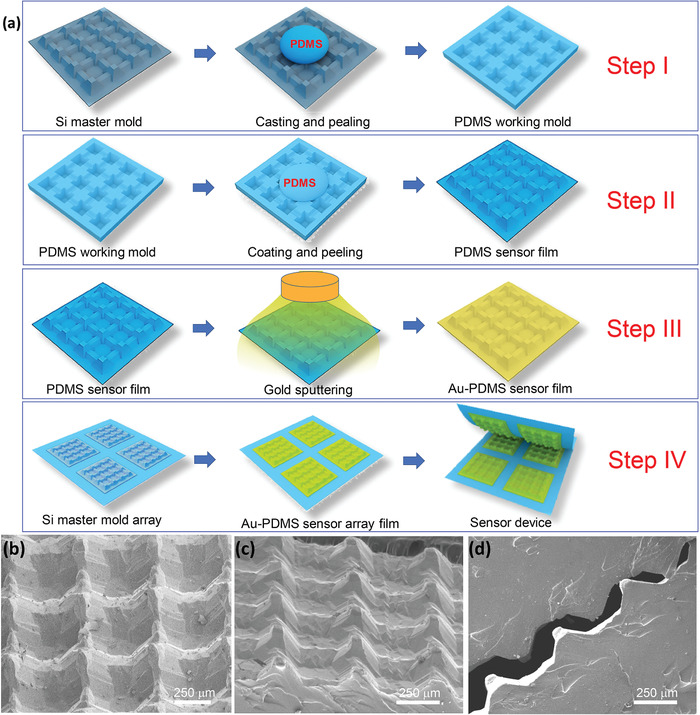
a) Schematic illustration for the fabrication process of a flexible supersensitive pressure sensor. b,c) Scanning electron microscope images of microstructured PDMS film with different angles and d) interlocked PDMS substrates.

**Figure** **1**a shows the fabrication process for the flexible piezoresistive pressure sensor with ultrahigh sensitivity schematically. The PWGM flexible PDMS films were golden‐sputtered to generate conductive layers before being assembled into an interlocked pressure sensor.

Scanning electron microscope (SEM) images of the PDMS working mold prepared in Step I may be found in Figures S3 and S1a–d of the Supporting Information. They show that the working mold possesses a grid structure composed of funnel‐like caves and grooves, which is the reverse of the microstructure of the silicon template. Each funnel‐like cave has a bowl‐like bottom, is located at a cross‐point of the *x* and *y*‐axis grooves, is much deeper than the grooves, and forms part of a square dot matrix. The morphology and depth of the funnel‐like caves and grooves matches the reversed morphology of the dome‐topped pyramids and strengthening walls of the silicon microstructured template without any distortion. A more detailed description of the working mold can be found in Section S3 (Supporting Information).

Figure 1b–d shows the SEM images of typical gold‐coated PWGM flexible PDMS film prepared through the secondary molding process using the PDMS working mold as the template. The microstructure and detailed description of the master mole for preparing the PWGM flexible PDMS film can be seen in Section S2 (Supporting Information). The PWGM flexible PDMS film precisely replicates the PWGM morphology of the silicon master mold. Figure 1b,c clearly shows the square pyramids with round tops, connected by strengthening walls to form the PWGM. Comparing Figure 1b,c; and Figure S2 (Supporting Information), we can find that the pyramids possess the greatest height (555 µm), the *x*‐axis strengthening wall has a smaller height (254 µm), and the *y*‐axis strength wall has the smallest (226 µm). Figure 1d shows a cross section of a sensing device formed by stacking two gold‐coated PWGM flexible PDMS films face‐to‐face. The top of the pyramids on the surface of one film are inserted into the basin surrounded by the strengthening wall of the opposite film, resulting in an interlocked contact mode. The well‐designed morphology and microstructure of the sensing film and the interlocked contact mode are unlike the morphologies and microstructures previously reported in the literature.^[^ ^20^, ^22^, ^24^, ^32^, ^33^, ^34^, ^35^, ^36^, ^37^ ^]^ The multiheight hierarchical microstructure of the different structural components and the flexible characteristics of PDMS as a material should endow the film with a special pressure‐sensing performance.

### 2.2 Sensing Performance Under Pressure

The pressure sensing device integrated by stacking a pair of gold‐coated PWGM flexible PDMS films face‐to‐face was built to assess the sensitivity and repeatability of the sensor materials (Figure S5, Supporting Information). Prior to the systemic investigation of the film's sensing property, a qualitative assessment of the sensitivity and repeatability of measurements by the device was performed by intermittently putting a finger on the device, then withdrawing it (Figure S5a, Supporting Information). Figure S5b (Supporting Information) illustrates the current response of the pressure sensor under nonconsecutive finger touches with an applied voltage of 1 V. As shown in the figure, the measured current under a gentle touch was more than several milliamperes After the finger was withdrawn from the pressure sensor, the current has a significant change in a short time. The rapid and drastic change of the current indicates that the sensor has a high sensitivity and excellent recovery.

To assess the sensing property of the device quantitatively, the current variation with pressure was measured. With 1 V of applied voltage, a gradually increasing pressure was loaded vertically on the device, and the real‐time current was recorded simultaneously. The resulting current versus loading force and loading force versus time curves as well as the integrated current versus loading force curves are shown in Figure S6 (Supporting Information). From Figure S6 (Supporting Information), we can see that, at times when there is a stepped increase of the pressure loaded on the device, there is also a stepped increase of the measured current.

**Figure 2:**
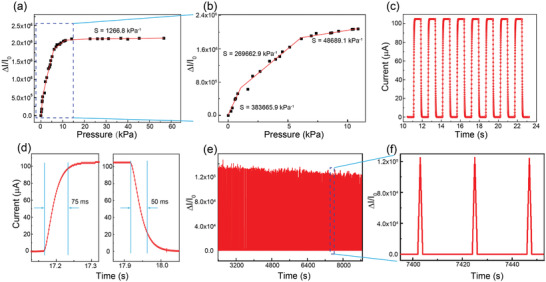
Sensing properties of the flexible pressure sensor. a) Relative change in current as a function of pressure in the range from 0 Pa to 60 kPa. b) High‐resolution curve at low pressure range, 0–11 kPa. c) Current response of the pressure sensor for eight cycles with applied pressure of 10 Pa. d) Response and recovery time of the pressure sensor under a loading/unloading pressure of 10 Pa. e) Durability of the pressure sensor tested by repeatedly loading and unloading (total duration was 10 000 s) with an applied pressure of ≈0.26 kPa. f) The recorded current variations from 7401 to 7448 s.

Based on the result in Figure S6 (Supporting Information), the relative current variation Δ*I*/*I*_0_ at different loaded pressures was calculated. **Figure** **2**a shows that relative current variation increases with loaded pressure, but that the slope of the curve is different in different parts of the pressure range. The sensitivity of the pressure sensor can be calculated from the data in Figure 2a. The sensitivity *S* is defined as the slope of the Δ*I*/*I*_0_–*P* curve

(1) $$S=\Delta \left(\Delta I/I_{0}\right)/\Delta P$$

where *I*_0_ is the initial current without pressure loading, Δ*I* is (*I*–*I*_0_), the difference between the data of the instantaneous current under a regular pressure and the initial current, and Δ*P* is the variation in loaded pressure.^[^ ^12^ ^]^

As shown in Figure 2a, the curve of Δ*I*/*I*_0_ versus *P* is not a straight line, but can be divided into four parts according to its different slopes at different pressure range. The calculated sensitivity for these four regimes is labeled in Figure 2a,b. As shown in the figures, the sensitivity of the device is 383 665.9 kPa^−1^ in the low pressure range (0–1.6 kPa), and 269 662.9 kPa^−1^ in the medium pressure range (1.6–6 kPa). In the high pressure range (6.1–11 kPa), the sensitivity is 48 689.1 kPa^−1^. Even in the very high pressure range of 11–56 kPa, the sensitivity of the sensor remains as high as 1266.8 kPa^−1^.

Response time, repeatability, and mechanical durability are also very important factors for a pressure sensor in practical applications.^[^ ^41^ ^]^ The response time for the loading and releasing pressure, which were defined as the time required to reach 90% of the maximum current and the time when the declined current reach to 1/*e* of the maximum current, respectively (where *e* is the natural constant), was assessed by recording the pressure‐induced current every 2 ms (shown in Figure 2c,d). From the figures, we can see that the device responds quickly to the loading and releasing of the pressure. The recorded current‐time peaks have the same shape and largest current, demonstrating a precisely producible pressure response. From Figure 2d, the response times of the pressure sensor are 75 and 50 ms for the loading and releasing responses, respectively.

To test the mechanical durability of the device, a pulsed pressure with a load of 0.26 kPa and a period of 23 s was repeatedly applied, and the current was measured (with an applied voltage of 1 V). As shown in Figure 2e, the relative current difference, Δ*I*/*I*_0_, stayed at ≈120 000 in every round of the loading without pronounced change. Even after 1000 loading/unloading cycles, the value of Δ*I*/*I*_0_ was almost the same as in the first round, indicating that the golden layer was not damaged by fast and repeated friction due to the strong interaction between PMDS film and golden particles in golden layer as shown in Figure S7 (Supporting Information). Figure 2f shows that the current pulses from the loading/unloading time of 7401–7448 s had exactly the same amplitude and peak morphology, confirming the high repeatability and durability of the pressure sensor.

To assess pressure detection limitations of the pressure sensor device, layers of square weighting paper of 2.5 × 2.5 cm were used as loading objects for the sensor to detect. As shown in Figure S8 (Supporting Information), the measured current of the sensor increased with the number of layers of paper. The current from the pressure of a single layer of paper is ≈40 µA, and several rounds of detection indicate good repeatability. The calculated pressure limit found by taking one piece of paper as the lowest loading pressure is 0.25 Pa. Even with a very small loading pressure, the device is still highly responsive and retains very good repeatability. More detailed results and discussions can be found in Section S7 (Supporting Information).

To compare the performance of the gold‐coated PWGM flexible PDMS film‐based pressure sensor with that of other piezoresistive sensors, the main properties of the pressure sensors reported in the literature and those of the pressure sensor in this work are listed in **Table** **1**. From the table, we can see that the sensitivity of most piezoresistive pressure sensors ranges from several tens to one thousand, and the highest sensitivity in any previous work is ≈10 000 kPa^−1^. This is much lower than the sensor sensitivity in our work, which is ≈380 000 kPa^−1^.

**Table 1:** Summary of piezoresistive pressure sensors

Structure	Sensitivity [kPa^−1^]	Testing pressure	Response time [ms]	Sensing limit [Pa]
Mimosa‐inspired micropatterns^[^ ^42^ ^]^	50.17	<780.3 Pa	<20	10.4
PEDOT:PSS/PUD^[^ ^22^ ^]^	56.8	<6 kPa	200	23
Interlocked Au‐coated PDMS microdomes^[^ ^34^ ^]^	196	<100 kPa	26	0.5
Conical frustum‐like PDMS/Ag and PI/Au^[^ ^37^ ^]^	259.32	<54 kPa	≈0.2	0.36
PEDOT:PSS‐coated Irregular hemisphere micropattern^[^ ^32^ ^]^	851	<20 kPa	0.15	34
PPy/PDMS micropyramids^[^ ^43^ ^]^	1907.2	<100 Pa	0.05	0.075
Thin SWCNT coated PDMS with micropyramids^[^ ^44^ ^]^	8655.6	<12 kPa	<4	7.3
Interlocked MWNT/PDMS composite with microdomes^[^ ^36^ ^]^	90657	<26 kPa	12	0.09
Au‐coated PDMS film (this study)	383 665.9	<60 kPa	75	0.25

Normally, the sensitivity of a piezoresistive pressure sensor become very low in the high pressure range, and almost all the best sensitivity results were obtained at very low pressures. Although the testing pressure in the literature can be as high as several hundred Pa, the reported sensitivity in this regime is just several tens, limiting the usefulness of such sensors for precise pressure measurement.^[^ ^22^, ^32^, ^34^, ^36^, ^39^, ^42^, ^43^, ^44^ ^]^ However, in our work, even when the pressure is higher than 10 kPa, the sensitivity stays at ≈50 000 kPa^−1^, very much better than in previously published work. The response time of our sensor is 75 ms, which can fulfill most practical requirements of pressure measurement, and is much shorter than in most published work. The pressure‐detection limit in our work is 0.25 Pa (due to the lack of more effective pressure loading method); this still takes third place among all the reported pressure limit data.

Summarizing the above results and analysis, the pressure sensor device based on the gold‐coated PWGM flexible PDMS film possesses ultrahigh sensitivity within different pressure sensor (as high as 380 000 kPa^−1^), a very broad working pressure range (from 0.25 Pa to 60 kPa), a short response‐recovery time (75 ms), an outstanding minimum detectable pressure (≈0.25 Pa), and long‐term cyclic stability (no pronounced sensitivity drop after 1000 cycles). Despite the above characteristics, the measured pressure sensitivity of the gold‐coated PWGM flexible PDMS film‐based pressure sensor is nearly same as the as‐prepared sensor device, which can ensure the stability of the sensor device in practical applications.

The ultrahigh sensitivity of the pressure sensor originates in the extra conducting paths formed by the contact of the dome‐like heads of the rectangular pyramids with the basins between the two perpendicular sets of strengthening walls, as well as the contact between the walls themselves.^[^ ^32^, ^37^ ^]^ The golden layer covering the sensor's microstructured surfaces also contributes to the sensitivity because of its rough surfaces formed by golden particles (Figure S7, Supporting Information) and the existence of cracks (Figure S9, Supporting Information), which act like porous spacer.^[^ ^45^ ^]^ So does the low initial current *I*_0_ (0.00 445 µA), which is a result of the small dome‐basin contact area in the unloaded state.^[^ ^36^, ^37^ ^]^ The sensitivity of the pressure sensor remained roughly linear at different pressure ranges, indicating that the deformation of the engineered microstructured pressure sensor is far from its saturation point even in the relatively high pressure range. Because of these features, the pressure sensor seems highly practical for the quantitative detection of various human activities when used as e‐skin, wearable electronics, or a health monitoring device.

### 2.3 Sensing Mechanism

**Figure 3:**
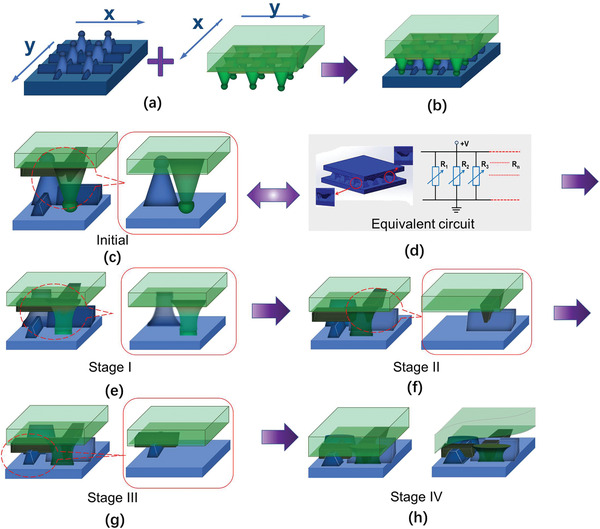
Schematic diagram of the deformation mechanism of the PWGM flexible PDMS sensor device. a) The microstructure of a pair of gold‐coated PWGM flexible PDMS films. A pair of films is assembled face‐to‐face with the *x* and *y* axes of the bottom film perpendicular to those of the up film. b) The resulting interlocked contact pressure sensor. c) The initial state: one unit of a pair of the microstructure, with the dome‐like top of each pyramid touching the bottom of the blanked area of the microstructured film. d) The interlocked contact mode, and its equivalent circuit with multiple resistors. e) Stage I: the dome‐like tops of the pyramids begin to be compressed under low‐pressure loading. f) Stage II: the pyramid is severely distorted by higher pressure loading, and the *x*‐axis strengthening walls begin to be compressed. g) Stage III: the pyramids and the *x*‐axis strengthening wall are both greatly distorted, and the *y*‐axis strengthening walls start to be compressed. h) Stage IV: with very high pressure loading, all the microstructures are deformed at the same time, and the deformation becomes harder.

The ultrasensitivity of the sensor device in this work over a wide range of pressures is a result of the unique pyramid‐wall grid microstructure of the sensing material. As mentioned in Section 2.2, the PWGM film is comprised of uniformly distributed dome‐topped square pyramids connected with strengthening walls parallel to the *x*‐ and *y*‐axes, with a characteristic height hierarchy for the different microstructure components. The dome‐topped pyramids are twice the height of the strengthening walls parallel to the *x*‐axis, which are themselves considerably higher than those parallel to the *y*‐axis. This engineered hierarchical flexible PDMS structure endows the PWGW sensor device with ultrahigh sensitivity over a broad pressure range, and with good repeatability. The proposed sensing mechanism is shown in **Figure** **3**.

Figure 3a shows the face‐to‐face packaging of two gold‐coated PWGM flexible PDMS films with electrodes. The sensor structure can be seen in Figure 3b. Figure 3c shows the initial stage of the sensor: the dome‐topped pyramids, inserted into the basins surrounded by the strengthening walls parallel to the *x*‐ and *y*‐axes, form an interlocked‐mode sensing device. As shown in Figure 3d, this is equivalent to a circuit with multiple resistors connected in parallel.^[^ ^41^ ^]^ The total resistance *R* of n resistors in parallel is given by 1/*R* = 1/*R*_1_ +1/*R*_2_ +… +1/*R*_n_. The current measured on the sensing device can be obtained from the equation *I* = *U*/*R*, where *I* is the current, *U* is the voltage applied to the device (1 V in this work), and *R* is the total contact resistance between the two sensing films. Evidently, the resistance for each individual contact point is inversely proportional to the contact area at that point, which in turn depends on the local deformation of the nanostructures on the surface of the sensing films. In general, more deformation results in more contact area, and thus in less resistance (and higher current, when the applied voltage is fixed at 1 V). The hierarchical deformation process can be divided into four stages, illustrated in Figure 3c–g.

##### Stage I: Deformation of Dome‐Topped Pyramids

When a very small pressure is loaded on the sensor, the deformation enters Stage I (Figure 3d). At this stage, deformation occurs only at the dome‐like heads of the pyramids of the nanostructured films. Because the round domes can very easily be flattened, the contact area can increase rapidly with the loaded pressure. Therefore, in Stage I, the sensor device possesses an ultrasensitive performance. Observationally, in the low pressure range (0–1.6 kPa), the pressure sensitivity reaches the extremely high value 383 665.9 kPa^−1^.

With increasing pressure, the dome‐like top gradually becomes flat, and the pyramids themselves shorten. At some point, the deformed pyramids become so short that the strengthening walls parallel to the *x*‐axis come into contact, and the deformation of the nanostructures on the films enters Stage II.

##### Stage II: Deformation of *x*‐Axis Strengthening Walls

In Stage II (shown in Figure 3e), the pairs of crossed *x*‐axis strengthening walls make contact and compress each other under the pressure loading. This is the principal contributing factor in the increase of the contact area (and thus of sensitivity) at this stage. However, because the pressure is also shared with the pyramids, which continue to deform at a low speed, the sensitivity is lower than in Stage I. This is consistent with the result that in the pressure range of 1.6–6 kPa, the sensitivity is as high as 269 662.9 kPa^−1^.

With further increase of the pressure loading, the deformation of the *x*‐axis strengthening walls continues, and their height keeps decreasing. When the pressure reaches a sufficiently high value, the deformation of the *x*‐axis strengthening wall approaches its limit, but the crossed *y*‐axis strengthening walls contact each other. Then, the deformation of the sensor enters Stage III.

##### Stage III: Deformation of *y*‐Axis Strengthening Walls

As is shown in Figure 3f, in Stage III, the deformation of the pair of *y*‐axis strengthening walls increases with the pressure loading. This results in contact area increase, and the measured current increases accordingly. In this stage, the pressure sensitivity is mainly derived from the deformation of the *y*‐axis strengthening walls. Because the deformed pyramids and the *x*‐axis strengthening walls share more pressure with less deformation, the pressure sensitivity is smaller than in Stages I and II. Nevertheless, because the contact area continues to increase due to the hierarchical height of the components of this uniquely engineered microstructure, the sensitivity in this stage is still much higher than that of other pressure sensors described in the literature: in the relatively high pressure range of 6.1–11 kPa, it is 48 689.1 kPa^−1^.

As the pressure loading continues to increase, the shortest structural components (the *y*‐axis strengthening walls) are continuously compressed, and reach their limit at a certain loaded pressure. This is the beginning of Stage IV.

##### Stage IV: Deformation of All Components on the Nanostructured Film

As shown in Figure 3g, in Stage IV, all the structural components support the loaded pressure at the same time, and the deformation occurs over the whole area of the pressure loading between the two films. In this situation, further deformation becomes very difficult, and the contact area increases very slowly. Therefore, at this stage, the sensitivity become much lower. However, because there are some gaps among the compressed structural components, deformation can still occur, and the contact area still increase, until all the gaps are filled. In this very high pressure range of 11–56 kPa, the sensitivity of the pressure sensor is still as high as 1266.8 kPa^−1^.

As the pressure continues to mount, the deformation of each microstructure tends to be stable. Therefore, the contact area would not change much. Maximum deformation corresponded to the state in which microstructure was not changing. The samples were same like the bulk PDMS.

To better understand the pressure loading process, the stress distribution on the surface of the nanostructured film under different pressures was simulated by finite element analysis. The simulation results and detailed discussion can be found in Figure S10, Supporting Information. The simulation results support the proposed hierarchical deformation mechanism for the sensing process.

### 2.4 Detection of Bending and Torsional Strains

**Figure 4:**
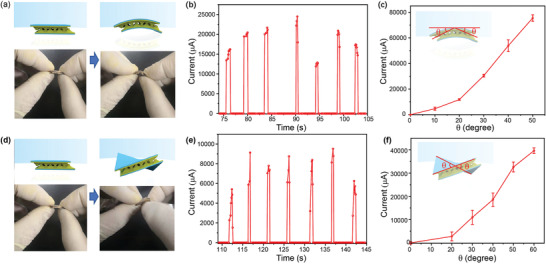
Flexible pressure sensor for detecting bending and torsional strain. a) Bending the sensor. Photographs show its flexibility in bending. b) Current response of the pressure sensor to the bending strain. c) Relationship between current and bending angle. d) Twisting the sensor. Photographs show its flexibility in twisting. e) Current response of the pressure sensor to the torsional strain. f) Relationship between current and torsional angle.

The flexibility of a pressure sensor, together with as its ability to sense bending and twisting, is very important for its application in wearable flexible electronics. The pressure sensor was therefore subjected to bending and torsional strains, and its real‐time current response under a voltage of 1 V was recorded. Based on the real‐time current response (shown in Figure S11, Supporting Information), we established the relationship between the current and the bending/torsional strain, which was expressed as the bending/torsional angle (**Figure** **4**c–f). As shown in Figure 4a,b, when a cyclical bending strain was applied, the pressure sensor yielded a prompt and repeatable current response with a roughly constant pulse value and rate (Figure 4b). The current was proportional to the received bending strain as the pressure sensor was bended to different angles (Figure 4c), indicating that the pressure sensor is able to detect bending strain competently. In addition, owing to its good flexibility, the pressure sensor could stand a large torsional strain. Its response to the torsional strain was stable, as shown in Figure 4d,e. When the sensor was twisted to different degree as shown in Figure 4f, the current changed with torsional angle proportionally. Based on its high sensitivity to bending and torsional strain, the pressure sensor of this work has potential applications in human wearable electronics.

### 2.5 Potential Applications of the PWGM Flexible PDMS Film‐Based Pressure Sensor

**Figure 5:**
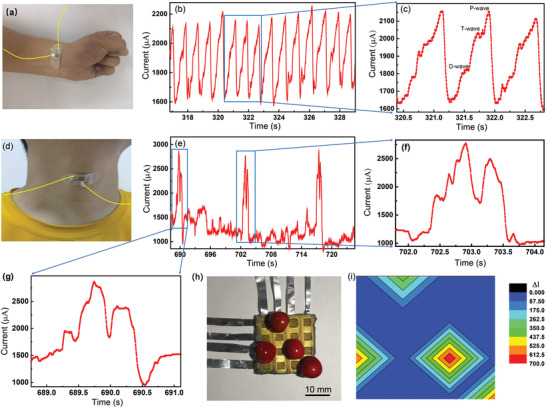
Applications of the flexible pressure sensor. a) Photograph of the pressure sensor attached to the author's wrist as a pulse monitor. b,c) Real‐time current output of pulse monitoring under a voltage of 1 V. d) Photograph of the pressure sensor attached to the author's throat to measure vocal cord vibration modes. e–g) Real‐time current output of vocal cord vibration monitoring for the utterance “Good morning.” h) Photograph of pressure sensor array with four red beans placed at different positions for mapping the pressure distribution. i) Current signal distribution under the pressure of the red beans.

Biomonitoring is the most popular application of flexible pressure sensors.^[^ ^46^, ^47^, ^48^, ^49^ ^]^ To demonstrate its biomonitoring capabilities, the pressure sensor was used to monitor the author's pulse and vocal cord vibration signals (**Figure** **5**). Figure 5a shows a photograph of the pressure sensor attached to the author's wrist, and Figure 5b,c shows the real‐time current output under a voltage of 1 V. Apparently, the pressure sensor produces strong periodic waveforms with personality (Figure 5b). Three peaks, representing the percussion wave (P‐wave), tidal wave (T‐wave), and diastolic wave (D‐wave),^[^ ^46^ ^]^ respectively, appear in the waveforms.^[^ ^36^ ^]^ The pulse rate was found to be 78 beats min^−1^, corresponding to the normal heartbeat of a young man. These results indicate that the pressure sensor can be used to monitor human biomedical signals.

Figure 5d shows a photograph of the pressure sensor attached to the author's throat to measure vocal cord vibrational modes. Figure 5e,f shows the real‐time current output for the author saying “Good morning” in English. As shown in Figure 5e,f, the pressure sensor produced strong current signals, with highly repeatable and recognizable peaks. By amplifying the peak signals of “good morning,” it was found that there were subtle changes in the waveforms of the phrase when the author said it with different tones. This observation suggests that the pressure sensor is able to “recognize” “talks” and even the tones of “talks.”

Furthermore, by placing 16 individual pressure sensors in a matrix‐like array, a composite pressure sensor with 4 × 4 pixels was fabricated as shown in Figure 5h. Four red beans were placed on the sensor array as shown in Figure 5h, inducing changes of current by the pressure of their weight. As shown in Figure 5h, each bean induced a series of current changes. The largest current change occurred just below the red beans on the sensor array; at greater distances from the bean locations, the changes gradually diminished to zero. This result shows that the pressure sensor has pixel resolution and performs like human skin in mapping external pressure; it thus has potential applications in both wearable electronics and intelligent robotics.

## 3 Conclusion

In summary, a flexible, wearable, extremely sensitive pressure sensor was designed and prepared using a four‐step casting method based on silicon molds with a unique pyramid‐wall grid microstructure. The structural components (dome‐topped pyramids and strengthening walls of different heights parallel to the *x*‐and *y*‐axes) were engineered to induce hierarchical deformation of the sensor materials. A four stage sequence of contact and deformation by the pyramids and walls was proposed to explain the extreme sensitivity of the device across a broad range of pressures. The flexible pressure sensor showed an ultrahigh sensitivity of 383 665.9, 269 662.9, and 48 689.1 kPa^−1^ in the pressure ranges less than 1.6, 1.6–6.1, and 6.1–11 kPa, respectively. It had a fast response time (within 75 ms), a low detection limit (less than 0.25 Pa), and excellent reproducibility over 1000 cycles. It exhibited a linear sensitivity over a very broad range from 0 to 60 kPa. Furthermore, the pressure sensor can detect bending and torsional strains, and measure the human pulse; it also has the capability of detecting pixels, as does human skin. These remarkable features suggest that the sensor has great potential for use in wearable electronics, humanoid robotics, wellness monitoring, and human–machine interfaces.

## 4 Experimental Section

##### Manufacture of the Silicon Master Mold

The silicon master mold was prepared by a standard microfabrication method. A commercial silicon wafer was used as the substrate. The manufacturing process included depositing a SiO_2_ layer by plasma‐enhanced chemical vapor deposition; preparing a photoresist mask; etching the SiO_2_; and plasma etching with SF_6_. The detailed experimental process is described in Section S1 (Supporting Information).

##### Fabrication of Microstructured PDMS Template and PDMS Film

The square silicon microneedle array of PWGM was separated from the PWGM, soaked in dimethyl formamide, and cleaned with ultrasonic cleaner three times. After being dried in an oven, the silicon microneedle array was fixed on a glass slide using AB glue. PDMS precursor (Sylgard 184, Dow Corning Co., Ltd) was mixed with the curing agent at a mass ratio of 10:1 under stirring and placed in a vacuum chamber at 25 °C for 10 min until bubbles disappeared.^[^ ^11^, ^18^ ^]^ The mixture of PDMS precursor and curing agent, i.e., uncured PDMS, was cast on the silicon microneedle array fixed to a glass slide. After being cured at 75 °C for 90 min in an oven, the PDMS film with concave microstructures was peeled off, sustained at room temperature for 2 h, and used as a PDMS template. The uncured PDMS was then cast on the PDMS template, cured at 75 °C for 60 min, and sustained at room temperature for 15 h. After that, the PDMS film with rectangular pyramids and strengthening walls was slowly peeled off and used as the primary unit of PDMS substrate in the pressure sensor.

##### Preparation of Pressure Sensor

The PDMS film with rectangular pyramids and strengthening walls was cut into a square shape and gold‐sputtered to fabricate conductive PDMS films. Before golden‐sputtering, the PDMS film was cleaned carefully using acetone, alcohol, and deionized water sequentially and then blow‐dried using nitrogen. The gold‐sputtering was performed on an ion sputtering apparatus (MC1000, HITACHI) at 10 mA for 5 min. Aluminum foil was cut into belts and fixed on the terminal of the gold‐sputtered PDMS film with silver paste. After that, the PDMS film was heated at 60 °C for 30 min to solidify the silver paste. Finally, the pressure sensor was assembled by fixing two PDMS films face‐to‐face and sealing them with adhesive to prevent external interference.

##### Characterization and Measurements

The microstructures of the silicon microneedle array of PWGM, the PDMS template prepared from the silicon microneedle array, and the PDMS film with rectangular pyramids and strengthening walls were all characterized using a scanning electron microscope (Regulus 8100, HITACHI) operated at 5 kV. The stress‐time curve of the pressure sensor was tested by using a pressure meter (HP‐2, HANDPI), and the current‐time characteristics of the sensor were determined using a SourceMeter (2400, Keithley) with a voltage of 1 V, under which the current measurement range was from 1 pA to 1 A. The sensor was connected to the testing system for cyclic loading/release tests at a constant speed of 45 times min^−1^, as shown in Figure S12 (Supporting Information). To determine the detection limit of the pressure sensor, 5 or 10 µL of water, which correspond to pressures of ≈5 or 10 Pa, respectively, were dropped onto the pressure sensor surface and absorbed with tissue. This was repeated for five cycles to observe the current response. To obtain the response and recovery time of the pressure sensor, the pressure meter and the pressure sensor were placed at a distance of 3 mm, so that when the pressure meter began to operate, the pressure sensor could be pressed as quickly as possible. For the application of the pressure sensor in biomonitoring, the pressure sensor was fixed to the author's wrist to test its real‐time current response to pulsating pressure (using the SourceMeter). To simulate human skin for feeling the position of an object, a pressure sensor with 4 × 4 pixels was fabricated by arranging 16 individual pressure sensors in a square array. The location information was collected by placing red beans on the sensor array at different positions to generate varied currents.

This research included human research participants (including some sensor experiments about detecting pulse and speaking). All written consent from all participants was obtained prior to the research.

## Conflict of Interest

The authors declare no conflict of interest.

## Supporting information

Supporting Information

## References

[1] Y. Zang , F. Zhang , C. Di , D. Zhu , Mater. Horiz. 2015, 2, 140.

[2] J. Li , R. Bao , J. Tao , Y. Peng , C. Pan , J. Mater. Chem. C 2018, 6, 11878.

[3] T. Yamada , Y. Hayamizu , Y. Yamamoto , Y. Yomogida , A. Izadi‐Najafabadi , D. N. Futaba , K. Hata , Nat. Nanotechnol. 2011, 6, 296. 21441912 10.1038/nnano.2011.36

[4] M. Y. Futaba , X. H. Huang , C. W. Ma , Y. J. Yang , J. Micromech. Microeng. 2009, 19, 115001.

[5] M. Amjadi , K. U. Kyung , I. Park , M. Sitti , Adv. Funct. Mater. 2016, 26, 1678.

[6] S. C. B. Mannsfeld , B. C. K. Tee , R. M. Stoltenberg , C. V. H. H. Chen , S. Barman , B. V. O. Muir , A. N. Sokolov , C. Reese , Z. Bao , Nat. Mater. 2010, 9, 859. 20835231 10.1038/nmat2834

[7] M. L. Hammock , A. Chortos , B. C. K. Tee , J. B. H. Tok , Z. Bao , Adv. Mater. 2013, 25, 5997. 24151185 10.1002/adma.201302240

[8] Y. Wang , L. Wang , T. Yang , X. Li , X. Zang , M. Zhu , K. Wang , D. Wu , H. Zhu , Adv. Funct. Mater. 2014, 24, 4666.

[9] S. H. Nam , P. J. Jeon , S. W. Min , Y. T. Lee , E. Y. Park , S. Im , Adv. Funct. Mater. 2014, 24, 4413.

[10] X. D. Wang , L. Dong , H. L. Zhang , R. M. Yu , C. F. Pan , Z. L. Wang , Adv. Sci. 2015, 2, 1500169. 10.1002/advs.201500169 PMC5115318 27980911

[11] T. Yang , D. Xie , Z. Li , H. Zhu , Mater. Sci. Eng., R 2017, 115, 1.

[12] X. Zhao , Q. Hua , R. Yu , Y. Zhang , C. Pan , Adv. Electron. Mater. 2015, 1, 1500142.

[13] Y. Wan , Y. Wang , C. F. Guo , Mater. Today Phys. 2017, 1, 61.

[14] C. Pang , G. Y. Lee , T. I. Kim , S. M. Kim , H. N. Kim , S. H. Ahn , K. Y. Suh , Nat. Mater. 2012, 11, 795. 22842511 10.1038/nmat3380

[15] Z. F. Chen , Z. Wang , X. M. Li , Y. X. Lin , N. Q. Luo , M. Z. Long , N. Zhao , J. B. Xu , ACS Nano 2017, 11, 4507. 28380292 10.1021/acsnano.6b08027

[16] F. R. Fan , L. Lin , G. Zhu , W. Wu , R. Zhang , Z. L. Wang , Nano Lett. 2012, 12, 3109. 22577731 10.1021/nl300988z

[17] S. Chen , K. Jiang , Z. Lou , D. Chen , G. Shen , Adv. Mater. Technol. 2017, 3, 1700248.

[18] H. B. Yao , J. Ge , C. F. Wang , X. Wang , W. Hu , Z. J. Zheng , Y. Ni , S. H. Yu , Adv. Mater. 2013, 25, 6692. 24027108 10.1002/adma.201303041

[19] N. Hu , Y. Karube , C. Yan , Z. Masuda , H. Fukunaga , Acta Mater. 2008, 56, 2929.

[20] J. Park , Y. Lee , J. Hong , M. Ha , Y. D. Jung , H. Lim , S. Y. Kim , H. Ko , ACS Nano 2014, 8, 4689. 24592988 10.1021/nn500441k

[21] J.‐S. Noh , Polymers 2016, 8, 123.

[22] C. L. Choong , M. B. Shim , B. S. Lee , S. Jeon , D. S. Ko , T. H. Kang , J. Bae , S. H. Lee , K. E. Byun , J. Im , Y. J. Jeong , C. E. Park , J. J. Park , U. I. Chung , Adv. Mater. 2014, 26, 3451. 24536023 10.1002/adma.201305182

[23] M. Park , J. Park , U. Jeong , Nano Today 2014, 9, 244.

[24] N. Khalili , X. Shen , H. E. Naguib , Soft Matter 2018, 14, 6912. 30095849 10.1039/c8sm00897c

[25] Y. Cao , T. Li , Y. Gu , H. Luo , S. Wang , T. Zhang , Small 2018, 14, 1703902. 10.1002/smll.201703902 29504238

[26] Q. Shao , Z. Niu , M. Hirtz , L. Jiang , Y. Liu , Z. Wang , X. Chen , Small 2014, 10, 1466. 24851243 10.1002/smll.201303601

[27] B. Zhu , Z. Niu , H. Wang , W. R. Leow , H. Wang , Y. Li , L. Zheng , J. Wei , F. Huo , X. Chen , Small 2014, 10, 3625. 24895228 10.1002/smll.201401207

[28] A. dos Santos , N. Pinela , P. Alves , R. Santos , E. Fortunato , R. Martins , H. Águas , R. Igreja , Adv. Electron. Mater. 2018, 4, 1800182.

[29] P. Nie , R. Wang , X. Xu , Y. Cheng , X. Wang , L. Shi , J. Sun , ACS Appl. Mater. Interfaces 2017, 9, 14911. 28402102 10.1021/acsami.7b01979

[30] M. Jian , K. Xia , Q. Wang , Z. Yin , H. Wang , C. Wang , H. Xie , M. Zhang , Y. Zhang , Adv. Funct. Mater. 2017, 27, 1606066.

[31] X. Wang , Y. Gu , Z. Xiong , Z. Cui , T. Zhang , Adv. Mater. 2014, 26, 1336. 24347340 10.1002/adma.201304248

[32] Z. Wang , S. Wang , J. Zeng , X. Ren , A. J. Y. Chee , B. Y. S. Yiu , W. C. Chung , Y. Yang , A. C. H. Yu , R. C. Roberts , A. C. O. Tsang , K. W. Chow , P. K. L. Chan , Small 2016, 12, 3827. 27280488 10.1002/smll.201601419

[33] Y. Zhang , Y. Hu , P. Zhu , F. Han , Y. Zhu , R. Sun , C. P. Wong , ACS Appl. Mater. Interfaces 2017, 9, 35968. 28952303 10.1021/acsami.7b09617

[34] Z. Wang , L. Zhang , J. Liu , H. Jiang , C. Li , Nanoscale 2018, 10, 10691. 29845159 10.1039/c8nr01495g

[35] H. Park , Y. R. Jeong , J. Yun , S. Y. Hong , S. Jin , S.‐J. Lee , G. Zi , J. S. Ha , ACS Nano 2015, 9, 9974. 26381467 10.1021/acsnano.5b03510

[36] J. Park , J. Kim , J. Hong , H. Lee , Y. Lee , S. Cho , S.‐W. Kim , J. J. Kim , S. Y. Kim , H. Ko , NPG Asia Mater. 2018, 10, 163.

[37] M. Chen , K. Li , G. Cheng , K. He , W. Li , D. Zhang , W. Li , Y. Feng , L. Wei , W. Li , G. Zhong , C. Yang , ACS Appl. Mater. Interfaces 2019, 11, 2551. 30576104 10.1021/acsami.8b20284

[38] Y. Pang , K. Zhang , Z. Yang , S. Jiang , Z. Ju , Y. Li , X. Wang , D. Wang , M. Jian , Y. Zhang , R. Liang , H. Tian , Y. Yang , T. L. Ren , ACS Nano 2018, 12, 2346. 29378401 10.1021/acsnano.7b07613

[39] T. Zhang , Z. Li , K. Li , X. Yang , Adv. Mater. Technol. 2019, 4, 1900679.

[40] W. Li , K. He , D. Zhang , N. Li , Y. Hou , G. Cheng , W. Li , F. Sui , Y. Dai , H. Luo , Y. Feng , L. Wei , W. Li , G. Zhong , M. Chen , C. Yang , ACS Appl. Energy Mater. 2019, 2, 2803.

[41] Z. Qiu , Y. Wan , W. Zhou , J. Yang , J. Yang , J. Huang , J. Zhang , Q. Liu , S. Huang , N. Bai , Z. Wu , W. Hong , H. Wang , C. F. Guo , Adv. Funct. Mater. 2018, 28, 1802343.

[42] B. Su , S. Gong , Z. Ma , L. W. Yap , W. Cheng , Small 2015, 11, 1886. 25504745 10.1002/smll.201403036

[43] H. Li , K. Wu , Z. Xu , Z. Wang , Y. Meng , L. Li , ACS Appl. Mater. Interfaces 2018, 10, 20826. 29847907 10.1021/acsami.8b03639

[44] Z. Huang , M. Gao , Z. Yan , T. Pan , S. A. Khan , Y. Zhang , H. Zhang , Y. Lin , Sen. Actuators, A 2017, 266, 345.

[45] Q. Tian , W. Yan , Y. Li , D. Ho , ACS Appl. Mater. Interfaces 2020, 12, 9710. 31992041 10.1021/acsami.9b18873

[46] T. Q. Trung , N. E. Lee , Adv. Mater. 2016, 28, 4338. 26840387 10.1002/adma.201504244

[47] X. Wang , Z. Liu , T. Zhang , Small 2017, 13, 1602790. 10.1002/smll.201602790 28306196

[48] S. Huang , Y. Liu , Y. Zhao , Z. Ren , C. F. Guo , Adv. Funct. Mater. 2018, 29, 1805924.

[49] W. A. D. M. Jayathilaka , K. Qi , Y. Qin , A. Chinnappan , W. Serrano‐García , C. Baskar , H. Wang , J. He , S. Cui , S. W. Thomas , S. Ramakrishna , Adv. Mater. 2019, 31, 1805921. 10.1002/adma.201805921 30589117

