# Supplementary material for: Engineered Microstructure Derived Hierarchical Deformation of Flexible Pressure Sensor Induces a Supersensitive Piezoresistive Property in Broad Pressure Range
Source: Adv Sci (Weinh). 2020 Aug 19;7(18):2000154. doi: 10.1002/advs.202000154 (PMC7509712; doi:10.1002/advs.202000154)
Supplement: Supplementary file 1 — Supporting Information [file ADVS-7-2000154-s001.pdf]

© 2020 Wiley-VCH GmbH

ADVANCED SCIENCE Open Access

Supporting Information

for *Adv. Sci.,* DOI: 10.1002/advs.202000154

Engineered Microstructure Derived Hierarchical Deformation of Flexible Pressure Sensor Induces a Supersensitive Piezoresistive Property in Broad Pressure Range

Gang Li, Duo Chen, Chenglong Li, Wenxia Liu, and Hong Liu**

Supporting Information

Engineered microstructure derived hierarchical deformation of flexible pressure sensor induces a supersensitive piezoresistive property in broad pressure range

Gang Li [a¶], Duo Chen [b¶], Chenglong Li [a], Wenxia Liu [a]*, Hong Liu [b,c*]

[a] State Key Laboratory of Biobased Materials and Green Papermaking, Qilu University of Technology, Shandong Academy of Science, Jinan, Shandong 250353, China

[b] Institute for Advanced Interdisciplinary Research, University of Jinan (iAIR), Jinan 250022, China

[c] State Key Laboratory of Crystal Materials, Shandong University, Jinan, 250100, P. R. China

[¶]Authors with equal contribution

Corresponding author: liuwenxia@qlu.edu.cn; hongliu@sdu.edu.cn

S1. Manufacture and microstructure of the silicon master mold

The silicon master mold was prepared by a typical microfabrication method. The main steps of the fabrication process of the pyramid-wall grid microstructureed silicon template are presented in **Figure S1**. A 4-inch silicon wafer with <100> crystallographic orientation, n-type 1–10 Ωcm resistivity was initially cleaned in piranha solution (H_2SO_4:H_2O_2 = 2:1) at 120°C for 20 min and then rinsed in DI water and spun dried (**Figure S1 a**). On the silicon wafer a 0.5 μm-thick SiO_2 was deposited at 300°C, from SiH_4 and N_2O, at a pressure of 700 mTorr and a power of 300 W using Plasma Lab80 plus equipment (**Figure 4b**). The positive photoresist SU8 was used for

thick photoresist lithography A photoresist mask using AZ4620 positive photoresist (from Microchem), with a thickness of 8µm, was used for the patterning of the SiO2-PECVD layer (**Figure S1 c**). The pattern is transferred to the SiO2 layer using a RIE etching system with CF_6 gases on an Adixen AMS100 SE (**Figure S1 d**). After the patterning of the oxide layer, the tips were generated using an isotropic RIE process (**Figure S1 e, f**) with SF_6 in an ICP- DRIE system. The process was optimized for a better controlled undercut, and with a depth-to-width following the designed 3D pattern.

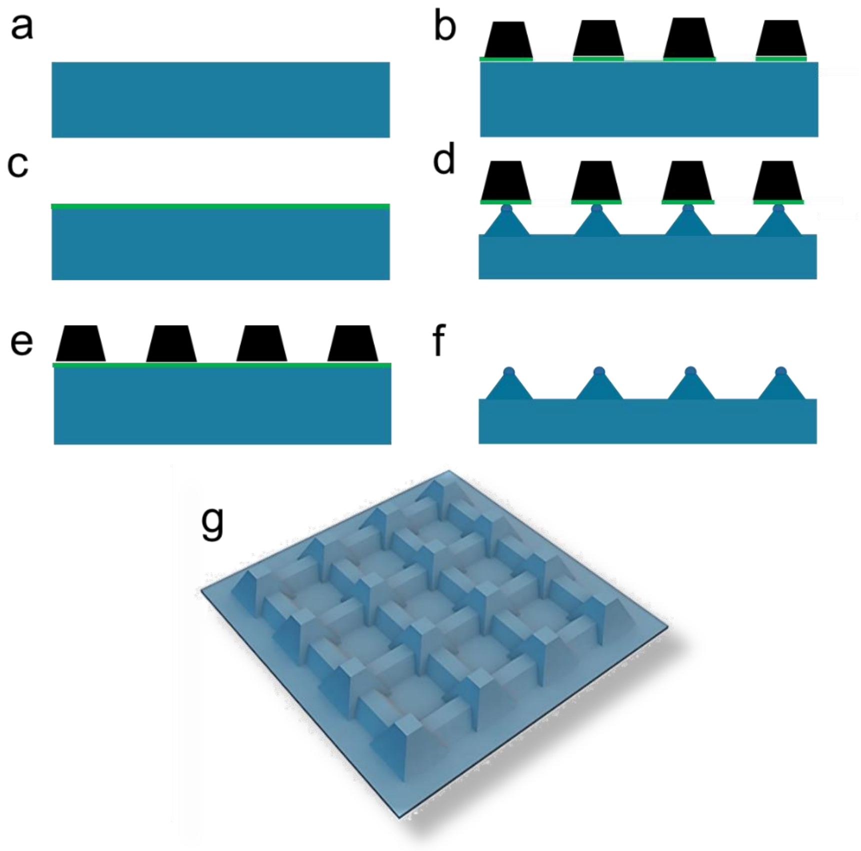

Figure S1 Illustration of preparation process of pyramid-wall grim nanostructure. a) silicon wafer; b) SiO_2 layer deposited by PECVD; c) Preparation of photoresist mask; d) Etching of SiO2, e) plasma etching with SF6.

S2. Microstructure of the pyramid-wall grid microstructured silicon master mold

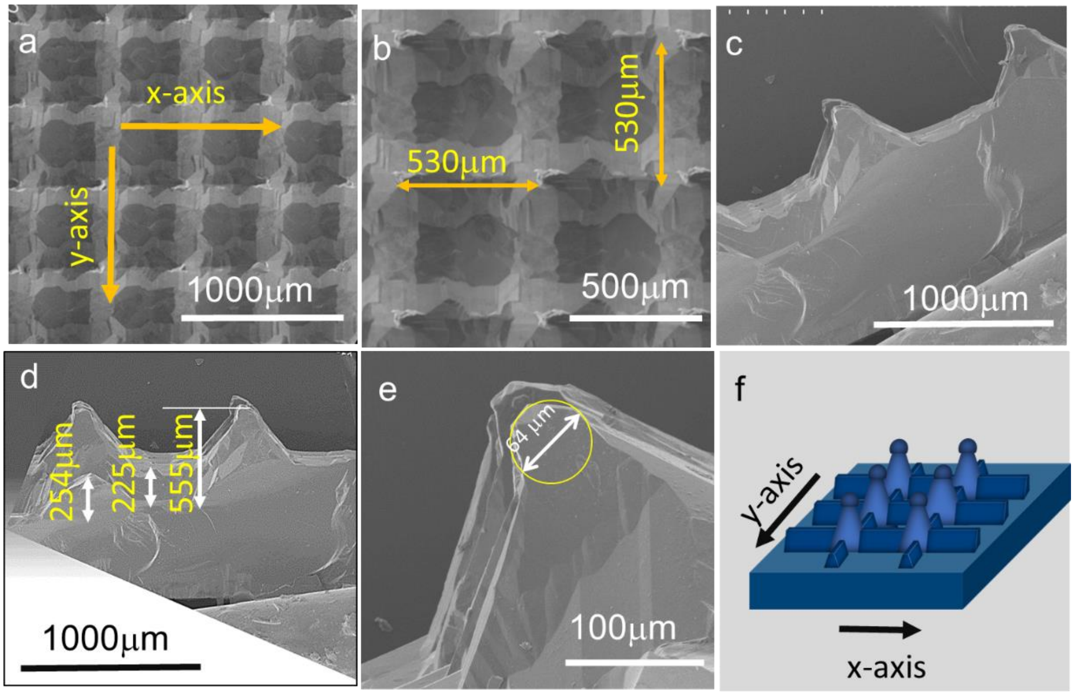

Figure S2 Microstructure of the silicon master mold. a) SEM images of top-viewed pyramid-wall grid microstructured silicon master mold; b) large magnification image of the master mold; c) SEM images of cross-section of the silicon master mold; and d) the heights of the dome-toped pyramid and the strengthening walls in x-axis and y-axis; e) the high resolution SEM image of the cross-section of the master mold, which shows the diameter of the dome-like top of the pyramid. f) illustration of the pyramid-wall grid microstructure of the silicon master mold.

Figure S2 shows the scanning electron microscopy (SEM) images of silicon master mold. As shown in **Figure S2a**, the silicon template is a typical 3-dimensioncal grid structure. The microstructure consists of square pyramids with dome-like tops (dome-toped pyramids) and strengthening walls which connected the pyramids in x and x

direction to form the grid structure (Pyramid-Wall Grid Microstructure, abbreviated as PWGM). As shown in **Figure S2b**, the dome-toped pyramids are uniformly distributed on surface of the silicon wafer as a square dot matrix. The distance between two neighborhood pyramids both in x- and y-axis are 530 μm (**Figure S2c**). The pyramid is a square shape, and the bottom side length of pyramids is 550μm. From **Figure S2d**, the measured height of the dome-toped pyramid, that of the strengthening wall in x-axis, ant of y-axis are 555 μm, 254μm, and 226μm, respectively. **Figure S2e** shows the amplified image of the pyramid, and the diameter of the dome-like top on the pyramid is about 65 μm, respectively. It is obvious that the height of the different structure components are different, the dome toped pyramid possesses largest height, the strengthening wall in x-axis have second height, and the strengthening wall in y-axis has the smallest height. This specifically hierarchical height microstructure is designed for the high-performance pressure sensor device.

S3. Microstructure of PDMS working mold

The PDMS working mold was prepared by mold-casting process from Step I. **FigureS3** shows the SEM image of the PDMS working mold. As shown in **Figure 3a**, the microstructure of the PDMS working mold is also a grid structure. The deepest funnel-like caves are distributed uniformly to form a square dot matrix, From **Figure S3 b**, we can find that the funnel-like caves have more deeper holes in the bottom. **Figure S 3c** shows that a round-shaped bowl-like pitch connected the bottom of the funnel, and from dome-tope funnel. The distance between two neighborhood funnel-like cave is 520, which consistent with the distance between two adjacent pyramids. The diameter of the round bottom of the funnel-like cave is about 65μm (**Figure S3d**), which is same as that of dome-like top of pyramid on the silicon master mold. The

funnel-like caves with a bowl-like bottom locate at the cross-point of x and y-axis grooves, which is much deeper than the grooves, and aligned to square dot matrix. The morphology and depth of funnel-like cave and grooves match well with the reversed morphology of dome-toped pyramids and the strengthen walls of the silicon microstructured template without any distortion.

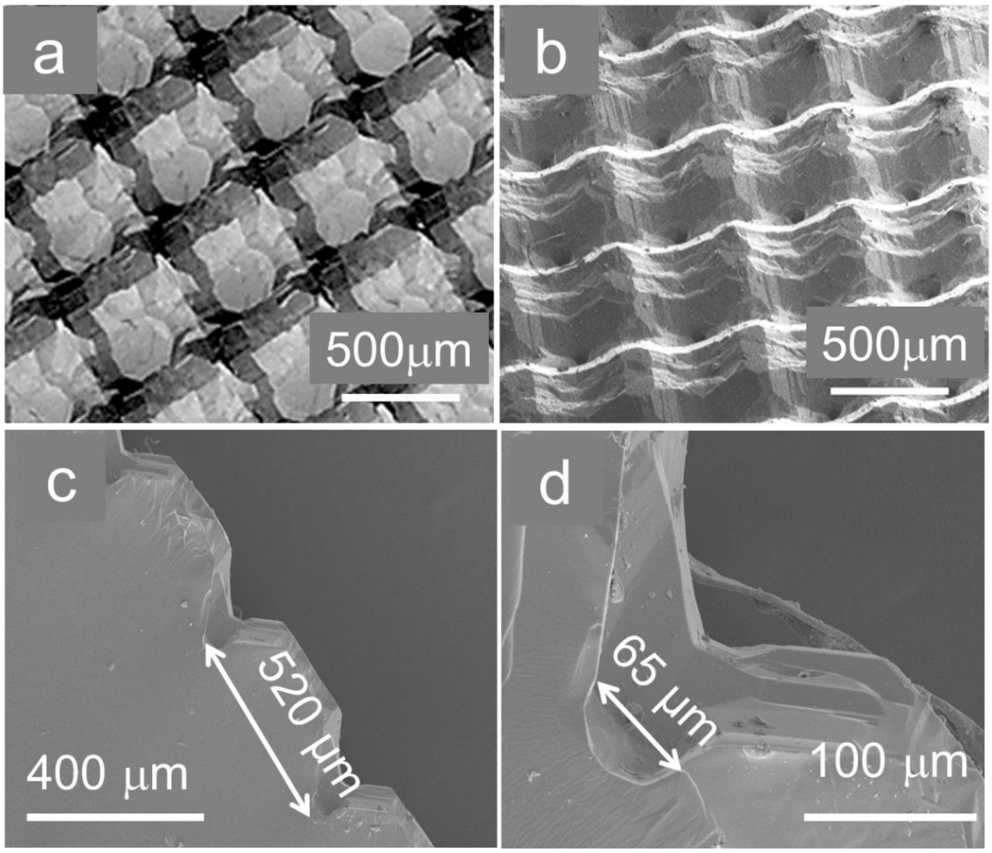

Figure S3 SEM images of the PDMS working mold. a) SEM image of top-view of the PDMS working mold. b) side-view of the PDMS working mold. c) SEM image of the PDMS working mold with low magnification. d) the hole copied from the dome-like tope of the pyramid of the silicon master mold.

The PDMS working mold is precisely coped the reversed morphology of the silicon master mold, and qualified to prepare the pressure sensor by a mold casting process.

S4. Structure of pressure sensor

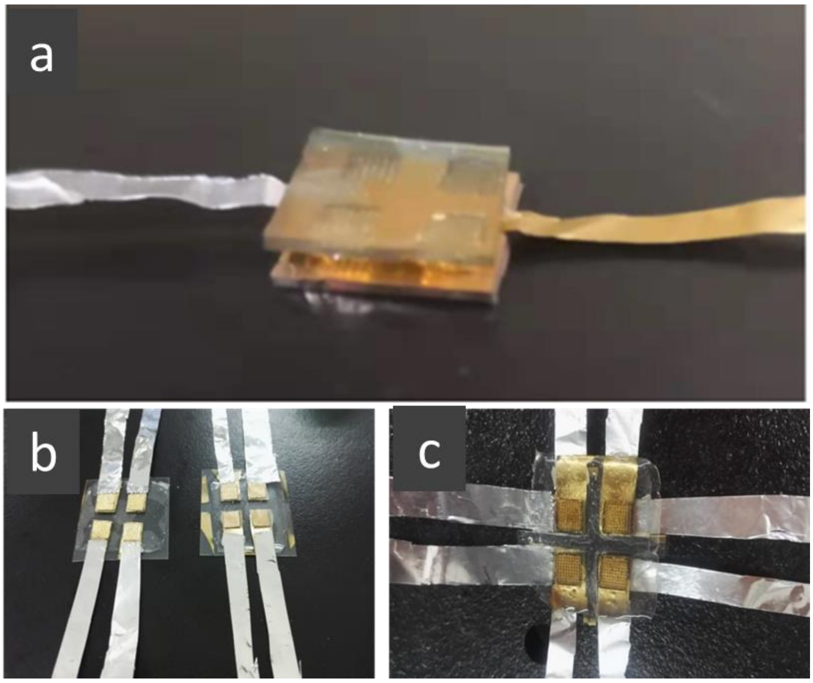

Figure S4 Photographs of a) a interlocked contact pressure sensor by face-to-face stacking a pair of gold coated PWGM flexible PDMS film. b) a 2*2 gold coated microstructured PDMS array with aluminum belts as electrodes. c) pressure sensor assembled by face-to-face staking two 2*2 coated PWGM flexible PDMS film .

The pressure sensor devices are shown as **Figure 4S**. At the back of the gold coated PWGM flexible PDMS film, an alumina belt was adhered to form an electrode. The pressure sensor was integrated by face-to-face stacking a pair of gold coated PWGM flexible PDMS films (**Figure S4a**). For getting 2×2 array, the 2×2 sensor films were prepared by the **Step IV**, in mold-casting process, which can be seen in **Figure 1**,

main text (**Figure S4b**). The sensing property was assessed by measuring electric current of the sensor device, and the applied voltage is fixed at 1V (**Figure S4c**)

S5. Qualitative assessment of the sensitivity and repeatability of the pressure device

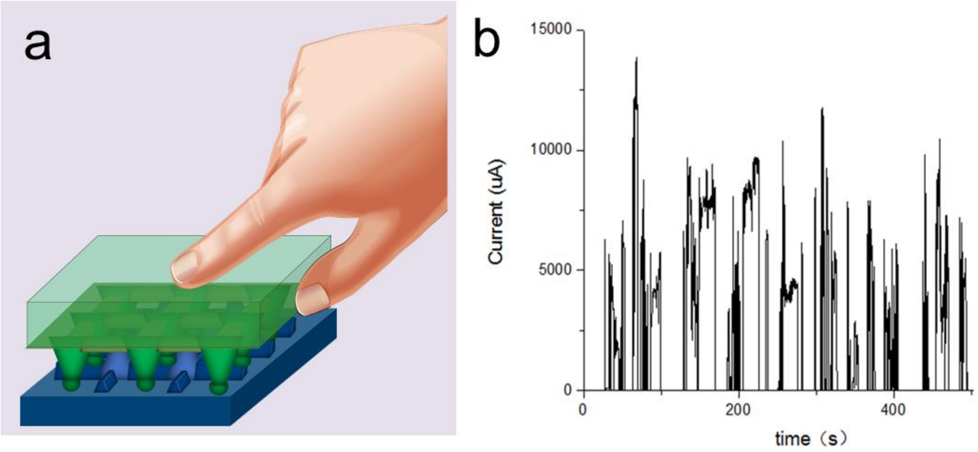

Figure S5 The schematic of the qualitative assessment of the sensitivity of the pressure sensor. a) Illustration of the finger pressing and releasing; b) Current response of the pressure sensor under inconsecutive finger touch. The applied voltage is 1 V.

Before the systemically study the sensing property of the PWGM flexible PDMS sensing film, a qualitative assessment of the sensitivity and repeatability of the pressure device was performed. The test was performed by gently touching and release of the finger. is more than several milliamperes (**Figure S5a**). **Figure S5b** illustrates the qualitative assessment of the sensitivity of the pressure sensor. The current response of the pressure sensor under inconsecutive finger touch with applied

voltage 1 V. As shown in **Figure S5**, the measured current by gently touch is more than several milliamperes. After withdrawing the finger from the pressure sensor, the current quickly reduced to approaching zero. The large current occurs and fast drops when the finger touches and releases, which indicates a high sensitivity and excellent recover property of the pressure sensor device. From the shape of the current-time curve, we can find that the response time is very short. Based on the qualitative assessment, the more detailed measurement of the sensor property of the sensor device.

S6. Real-time measurement of the current when the pressure gradually increase

The pressure applied on the sensor was calculated by dividing applied force with the contact area. The applied force on pressure sensor was gradually increased by increasing a small value each step in sensitivity calculation (Figure 2a and b). Therefore, the force is the accumulated value calculated by the sum of the force applied in each step one, i.e., in the loading process, the applied force is steppedly increased. For each step, there are an interval of 20~100 s to ensure the stability of the applied pressure and measured current signal, but there is no relaxing process to withdrawn the loaded pressure. The pressure data in Figures 2a and b were calculated from Figures S6a and b, which was plotted according to Figures S6c-d. As shown in Figure S6e and f, the maximum deformation occurred after the applied force reached to 16 N (corresponding to 90.91 kPa), after which the measured current is dropped with increasing the applied pressure. The maximum pressure applied in this work is thus limited to 60 kPa.

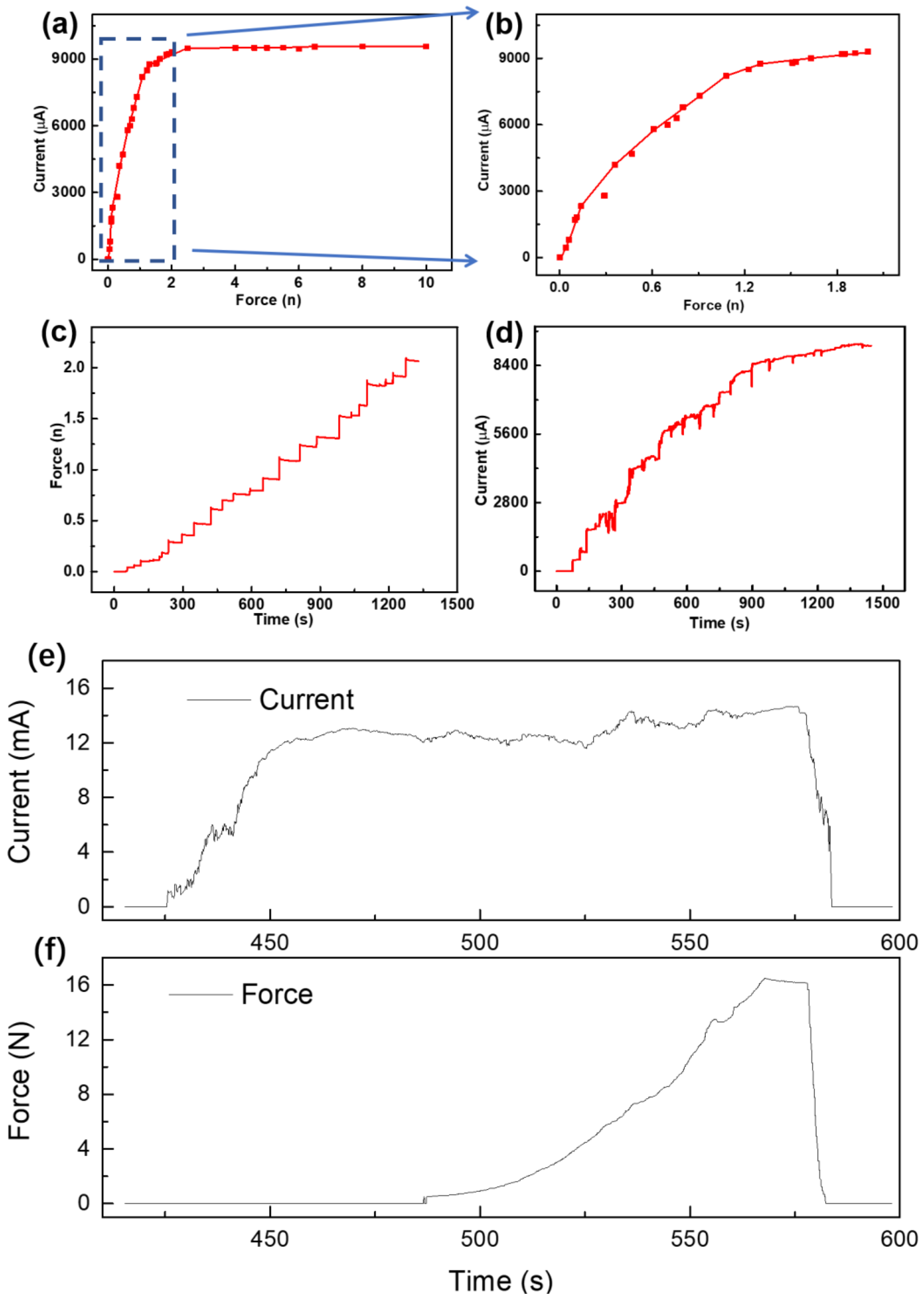

Figure S6 (a) The loading force-current curve. (b) The loading force-current curve at low pressure range, (c) the corresponding force vs. time curve and (d) the corresponding current vs. time curve. (e) current vs. time curve at high pressure rang and (f) the corresponding force vs. time curve at high pressure range.

S7. Cross-sectional SEM images of golden-sputtered PDMS film before and after the cyclic test

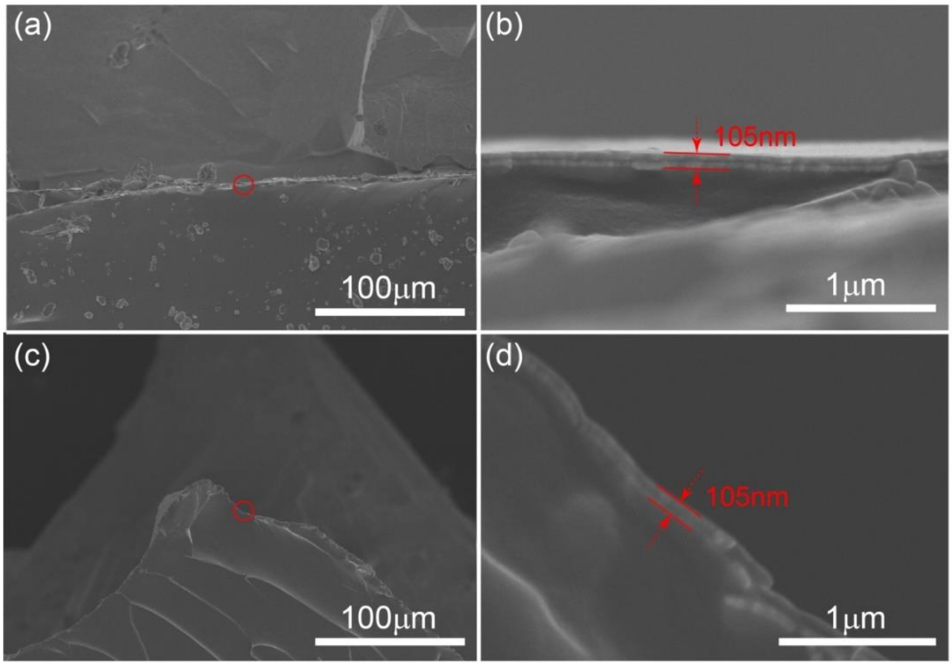

Figure S7 The cross-sectional SEM images of golden-sputtered PDMS film. (a) and (b) SEM images before cyclic test, (b) the amplified image of the region in red circle in (a). (c) and (d) the cross-sectional SEM images of golden-sputtered PDMS film after cyclic test. (d) the amplified image of the region in red circle in (c).

As shown in Figure S7, the golden layer was composed of golden particles and tightly bonded with PMDS film even after cyclic test.

S8. Sensing limitation the repeatability at small load assessment on the sensor device

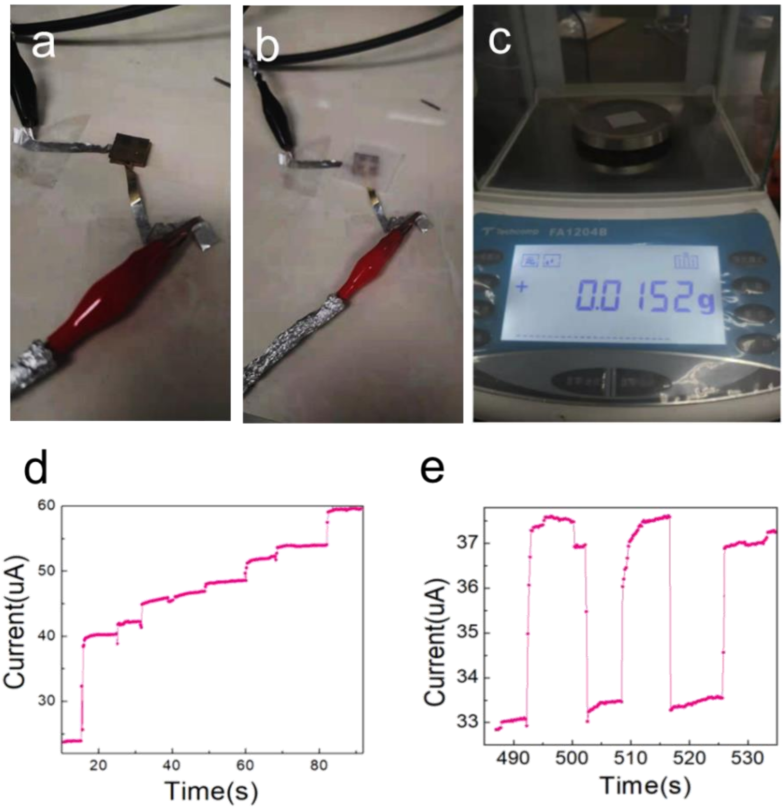

Figure S8 The sensing limitation test of the sensor device. a) photography of the sensor device. b) loading of a piece of weighting paper. c) the weight of the one layer of the paper. d) the current-time curve recorded on the device when the weighting paper stepped loaded on the sensor device. e) Current-time curve recorded on the sensor device when the weighting paper repeating loading and unloading process.

Figure S8 shows the assessment of the sensing limitation of the sensor device. The sensor device is shown in **Figure S8a**. To test the sensing limitation, a very thin weighting paper was taken as sample, shown in **Figure S8b**. One layer of paper was weighted, and the weight is 0.0152g (**Figure S8c**). The calculated pressure loaded on the surface of the pressure sensor for one layer of weighting paper is about 0.25Pa. As shown in **Figure S8d**, the current induced by loading the weighting paper increase stepped with layer-by-layer loading the weighting paper. The current measured on the

pressure sensor increase rapidly from the initial current 24μA to about 40μA, demonstrating that the sensor still possesses a very good sensitivity even the pressure loading is as low as 0.25Pa. To test the stability of the sensor at very low pressure loading, one piece of weighting paper was loaded and unloaded on the sensor, and the current of the sensor was recorded real-time. **Figure S8e** shows the current-time curve when the pressure loading and unloading took place on the sensor. The initial current derived from the contact resistance keep at 33μA, and the current at the pressure loading state is about 38μA. The reliability and the repeatability of the sensor are satisfied.

S9. Characterization of cracks on the Au sputtered surfaces

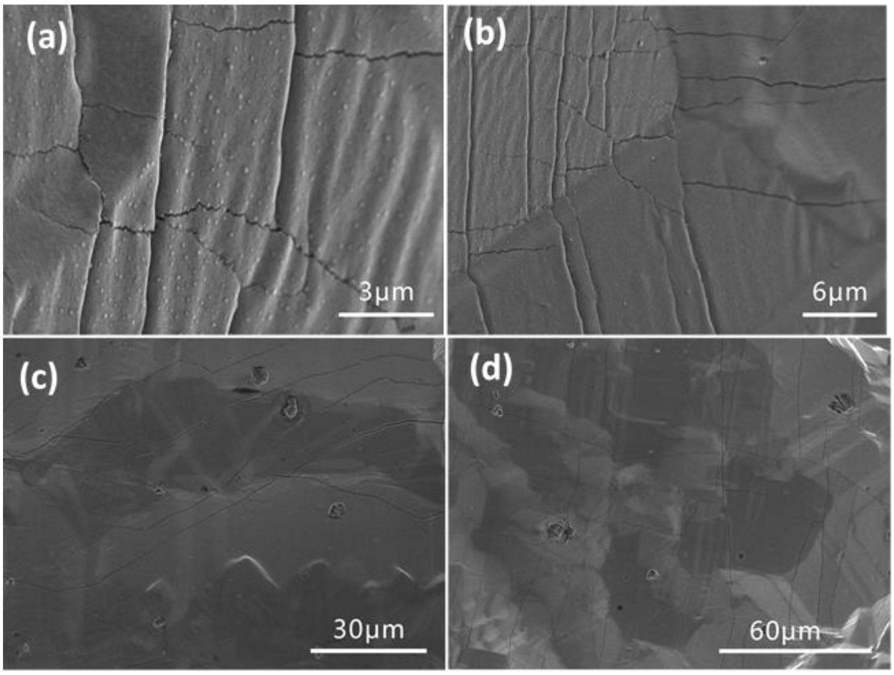

Figure S9 SEM images of Au-sputtered

As shown in Figure S9, gold layer is composed of golden particles and there are cracks, leading to a discontinuous golden layer. This largely increased the contact resistance of each contact point.

S10. Simulation of the stress distribution of the sensor film under the pressure loading

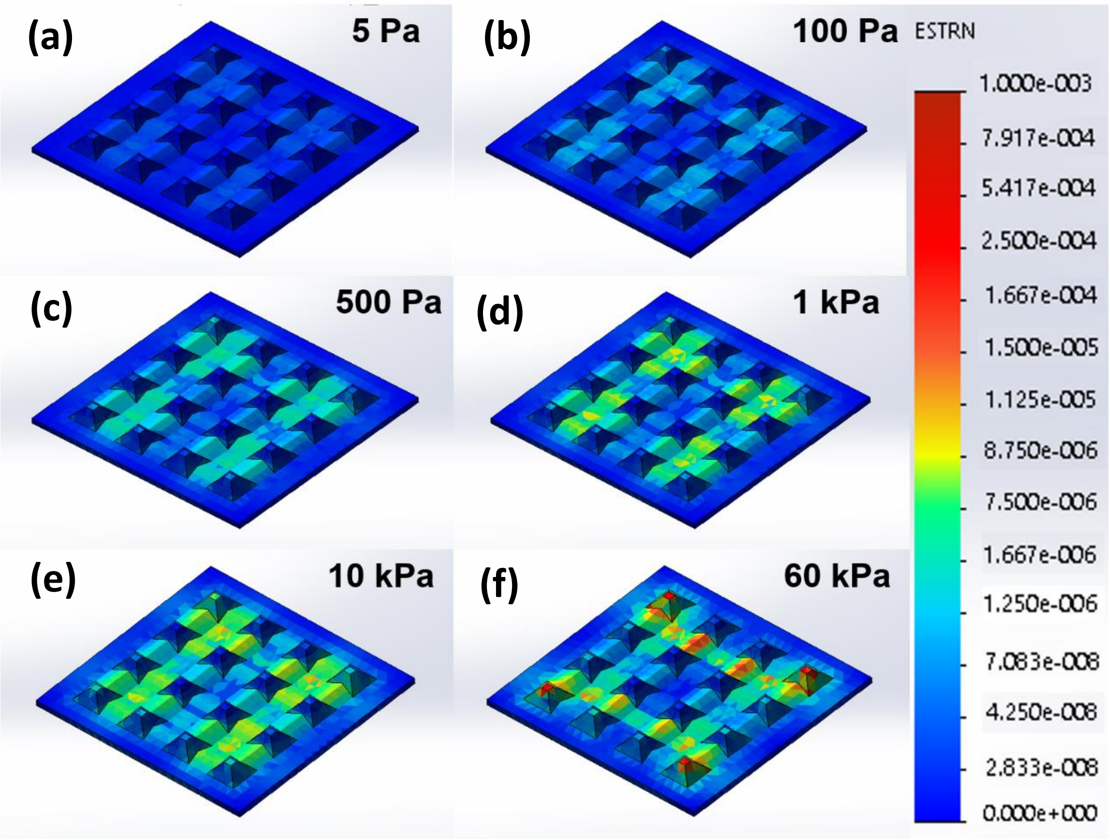

Figure S10 Stress distribution diagram of microstructured film under different applied pressures: a) 5 Pa pressure, b) 100 Pa pressure, c) 500 Pa pressure, d) 1 kPa pressure, e) 10 kPa pressure, f) 60 kPa pressure.

To better understand the pressure loading process, the stress distribution on the surface of the nanostructured film under the different pressure was simulated by Finite Element Analysis (FEA). A 3D model and simulations were performed using a

SOLIDWORKS & simulation (2016, Dassault Systèmes). The material properties of density and Poisson's ratio were assigned 1.12 g/cm3 and 0.28, respectively. To simplify the simulation process, the golden film was ignored. The microstructured film was uniform, and proper meshes were used for the contact area to increase the accuracy of the model. One of the designed microstructured films was fixed as a support, while the other one was loaded to simulate the contact process. Then the external pressures of 5 Pa, 100 Pa, 1 kPa, 10 kPa and 60 kPa were loaded on the model respectively. The color of stress is expressed as the variation of color as shown in Figure S10, Supporting Information. To better understand the pressure loading process, the stress distribution on the surface of the nanostructured film under the different pressure was simulated by Finite Element Analysis (FEA). The simulation results and the detailed discussion can be find in **Figure S10.**

The change of stress is expressed as the variation of color. More detailed FEA simulation is shown in **Figure S10**. In the model, the width and heights of the rectangular pyramids and their strengthening walls were selected in proportion based on the SEM images. Under a very low pressure such as 5 Pa, only the rectangular pyramids slightly contact with basins, producing a small stress on the tops of pyramids and the bottoms of basins (**Figure S10a**). With the increase in the pressure, the deformation of the pyramids and basins induces multi-directional distributed stress. When the deformation is increased to a certain extent, there are not much rooms for further increasing the contact area, then the strengthening walls with different directions start to contact. There are large stress produced on the strengthening walls (**Figure S10e**). The FEA result shows that the microstructured pressure sensor still sensitively responds when the pressure is beyond 10 kPa (**Figure S10f**). This simulation result is in accordance with the experimental data, but the stress

distribution diagram shows that the contact area has obviously covered the entire microstructure (**Figure S10f**). That is, the sensitivity is higher under low pressure than that under high pressure, i.e., decreases as pressure increases. This result is also similar to the $\Delta I/I_0$- ΔP curve shown in **Figure 2b**. The stress induces the contact area increase, and the measured current of the sensor increase. From the simulation results, we can find that the pressure induced stress can be divided into 4 stages, and in any stage, the deformation mainly causes by one different structure component, which is the reason that the sensor device possesses high sensitivity in a broad pressure range. The simulation results support the proposed hierarchical deformation mechanism of the sensing process.

S11. Current-time curves under different bending and twisting degrees

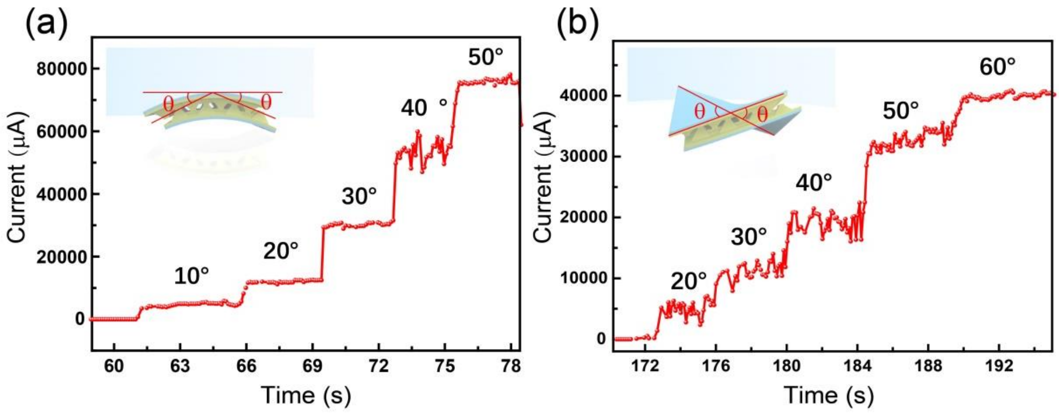

Figure S11 Current-time curves under different (a) bending angles and (b) torsional angles. The insets in (a, b) schematically show the flexibility of pressure sensor in bending and twisting, respectively.

Figure S11 shows the current-time curves under different bending and torsional angles. It was used to calculate the relationship between the measured current and the bending/torsional angles.

S12. The schematic diagram of the test system

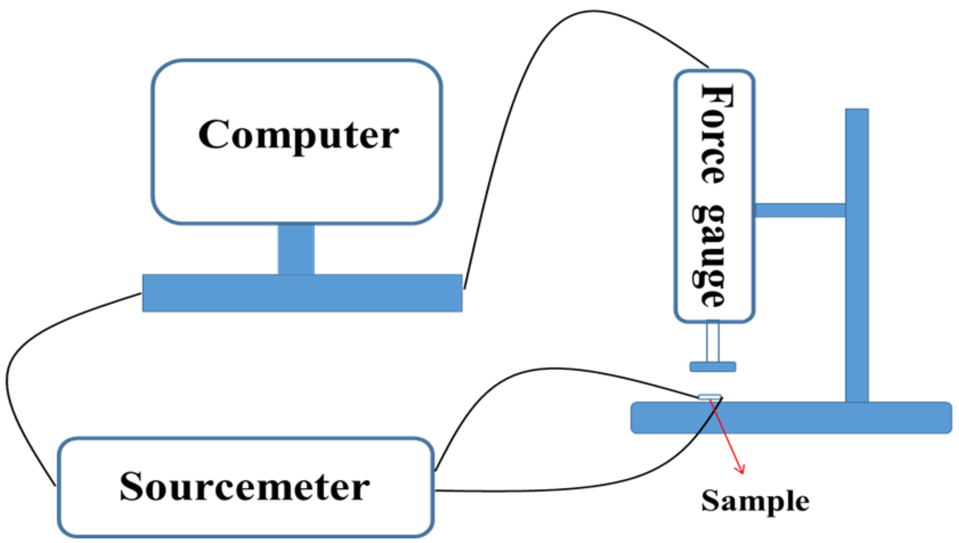

Figure S12 The schematic diagram of the whole test system.
